# Predictors of short and long term outcome in patellofemoral pain syndrome: a prospective longitudinal study

**DOI:** 10.1186/1471-2474-11-11

**Published:** 2010-01-19

**Authors:** Natalie J Collins, Kay M Crossley, Ross Darnell, Bill Vicenzino

**Affiliations:** 1 School of Health and Rehabilitation Sciences, The University of Queensland, Brisbane, Australia; 2 National ICT Australia, Melbourne School of Engineering, The University of Melbourne, Melbourne, Australia; 3 School of Physiotherapy, Faculty of Medicine, Dentistry and Health Sciences, The University of Melbourne, Melbourne, Australia

## Abstract

**Background:**

Patellofemoral pain syndrome (PFP) is a common musculoskeletal condition that has a tendency to become chronic and problematic in a proportion of affected individuals. The objective of this study was to identify prognostic factors that may have clinical utility in predicting poor outcome on measures of pain and function in individuals with PFP.

**Methods:**

A prospective follow-up study was conducted of 179 participants in a randomised clinical trial. Nine baseline factors (age, gender, body mass index, arch height, duration of knee pain, worst pain visual analogue scale, Kujala Patellofemoral Score (KPS), functional index questionnaire (FIQ), step down repetitions) were investigated for their prognostic ability on outcome assessed at six, 12 and 52 weeks (worst pain, KPS and FIQ). Factors with significant univariate associations were entered into multivariate linear regression models to identify a group of factors independently associated with poor outcome.

**Results:**

Long symptom duration was the most consistent predictor of poor outcome over 52 weeks rated on the KPS and the FIQ (β-0.07, 95% confidence interval -0.1 to -0.03, *p *< 0.000; and -0.02, -0.03 to -0.01, *p *< 0.000, respectively). Worse KPS at baseline was predictive of outcome at six, 12 and 52 weeks. Gender, body mass index and arch height were generally not associated with outcome (univariate analysis), while age, worst pain, FIQ and step downs were excluded during multivariate analyses.

**Conclusions:**

Patients presenting with PFP of long duration who score worse on the KPS have a poorer prognosis, irrespective of age, gender and morphometry. These results suggest that strategies aimed at preventing chronicity of more severe PFP may optimise prognosis.

## Background

Patellofemoral pain syndrome (PFP), or idiopathic pain arising from the anterior knee, is one of the most common musculoskeletal conditions affecting young active adults [1-6], and has a tendency to persist to some degree in a substantial proportion of affected individuals [7]. Prospective longitudinal studies have demonstrated the propensity towards chronicity of PFP in active populations (adolescent females [8], military personnel [9]). Identification of people who are likely to have persistent PFP will enable clinicians to provide patients with a more accurate picture of their prognosis [10], thereby facilitating negotiation of more realistic expectations of outcome and long term management [11].

Prognostic factors, such as demographic, diagnostic or co-morbid characteristics of an individual measured at baseline, are generally used to identify those with a greater likelihood of a poor outcome over time [12,13]. Several attempts have been made to determine prognostic indicators of outcome in PFP [14-17]; however, due to methodological issues it is difficult to draw valid conclusions applicable to the clinical context. For example, treatment was not accounted for in the design and analysis of any of these studies, thereby making it difficult to discern if it was the treatment or the derived prognostic factors that was responsible for the outcome. One study did not exclude volunteers with PFP of traumatic onset [14], while other studies utilised outcome measures not specific to PFP (e.g. Lysholm functional knee score [16,17]) or the clinic (e.g. laboratory-based tests [14]). Notwithstanding these shortcomings, the individual studies identified older age, greater height, and longer duration of symptoms as some prognostic factors, but none of these variables were found consistently across multiple studies.

On the basis of this, further high-quality research into the predictive ability of variables such as age, gender, body mass index (BMI), foot posture, duration of symptoms, pain severity and functional limitation is warranted. A systematic review found older age, higher baseline pain severity, longer pain duration and higher baseline disability to be consistent prognostic factors in musculoskeletal conditions [11]. The well-documented higher prevalence of PFP in females raises the question as to whether gender also influences prognosis [1,18]. Although a previous study failed to identify BMI as a significant prognostic factor [17], baseline data from a recent randomised clinical trial [19] revealed a trend towards the higher end of normal for healthy body weight. As BMI is modifiable through weight control, its identification as a prognostic factor would provide another intervention by which to improve the prognosis of PFP sufferers, and hence should be further investigated. Despite a postulated relationship between PFP and foot posture [20,21], the role of foot posture as a prognostic indicator has not been well investigated. Only one of the four previous prognostic studies investigated foot posture [17] using a measure of ankle hyperpronation during a bilateral squat (none 0-3°; mild 4-6°; clear > 6°). However, the reliability and validity of this measure was not reported. Considering the recent development of a reliable method of measuring the foot [22], the prognostic ability of foot posture in PFP should be investigated further.

The tendency for PFP to become persistent and problematic in some individuals has long term implications for participation in daily and work tasks as well as physical activity [7]. The current literature provides only preliminary data on which to determine the prognosis of sufferers of this condition. In order to address this shortfall, baseline measures of such variables as age, gender, BMI, foot posture, duration of symptoms, pain severity and functional limitation were evaluated for their influence on short and long term outcome. The objective of this study was to determine whether there are reliable factors with high clinical utility that are predictive of poor short term (six and 12 weeks) and longer term (52 weeks) outcome on clinical measures of pain and function in individuals with PFP.

## Methods

### Study design

A prospective follow-up study was conducted post-hoc using data from a previously reported single-blind, single centre, 12-month randomised clinical trial [19,23]. 179 participants with PFP were randomly assigned to a six-week treatment program of multimodal physiotherapy consisting of patellofemoral joint mobilisation, patellar taping, quadriceps muscle retraining and education (n = 45), prefabricated foot orthoses (n = 46), flat shoe inserts (n = 44), or foot orthoses plus physiotherapy (n = 44). Ethical approval was granted by The University of Queensland's medical research ethics committee, and all participants provided written informed consent.

### Participants

Participants were recruited from the greater Brisbane, Gold Coast and Toowoomba regions of Queensland, Australia between May 2004 and May 2006 through paid advertisements in print media, radio and television media, noticeboards, and referrals from practitioners. Volunteers underwent a clinical examination (including interview and physical examination) by an experienced physiotherapist, and were included if they fulfilled the following criteria [24]: (i) age 18 to 40 years; (ii) insidious onset of anterior knee or retropatellar pain greater than six weeks duration and provoked by at least two of: prolonged sitting or kneeling, squatting, running, hopping, or stair walking; (iii) tenderness on patellar palpation, or pain with step down or double leg squat; and (iv) pain over the previous week of at least 30 millimetres on a 100 millimetre visual analogue scale (VAS). Exclusion criteria were concomitant injury or pain from the hip or lumbar spine or other knee structures; previous knee surgery; patellofemoral instability; knee joint effusion; any foot condition that precluded use of foot orthoses; allergy to strapping tape; physiotherapy or foot orthoses within the previous year; or use of anti-inflammatory medication. Baseline characteristics of the participants are presented in Table 1.

**Table 1:** Baseline participant characteristics for the study cohort (n = 179); values are mean (SD) unless otherwise stated.

Characteristics	
Age (years)	29.3 (5.8)

Number (%) of females	100 (55.9)

Height (cm)	173 (9.5)

Weight (kg)	74.7 (18.6)

Body Mass Index (kg/m^2^)	24.8 (5.1)

Physical activity* (kcal/kg)	41 (7.3)

Bilateral knee pain: Number (%)	102 (57)

Duration of knee pain (months): Median (IQR)	28 (12-84)

Usual pain^	36.3 (16.6)

Worst pain^	60.5 (15.9)

Kujala Patellofemoral Score^†^	71.5 (9.8)

Functional index questionnaire^‡^	9.8 (2.1)

* Physical activity over the previous week questionnaire, total energy expended per day.

^ Pain measured on a 100 mm visual analogue scale; 0 mm = no pain, 100 mm = worst pain imaginable.

^† ^0-100 points; 100 = no disability.

^‡ ^0-16 points; 16 = no disability.

### Predictor factors and outcome assessment

A blinded assessor recorded potential prognostic indicators at baseline prior to randomisation. Nine factors were selected for investigation with respect to their prognostic ability. Participant characteristics included age, gender, BMI and weight bearing arch height, which was measured as the distance from the standing surface to the dorsal surface of the foot at a point halfway along the length of the foot [22]. PFP variables were represented by duration of knee pain and baseline scores of pain and function. Worst pain over the preceding week was rated on a 100 millimetre VAS, with the descriptors 'no pain' at the zero millimetre mark and 'worst pain imaginable' anchoring the 100 millimetre end [25]. The Kujala Patellofemoral Score (KPS) consists of 13 items with discrete categories related to limp, weight bearing, walking, stairs, squatting, running, jumping, prolonged sitting with flexed knees, pain, swelling, painful patellar movements, thigh muscle atrophy, and flexion deficiency [26]. Participants selected one response to each item that best described their knee condition, and individual items were summed to provide a final score where zero represented maximal disability and 100 represented no disability. The functional index questionnaire (FIQ) comprises eight questions regarding aggravating activities for PFP [27]. Participants rated their degree of difficulty performing each activity, with the sum of scores providing an overall score of disability ranging from zero (maximal disability) to 16 (no disability). Pain-free step down repetitions were performed on a 25 centimetre step in an order that continually loaded the nominated lower limb [24]. A metronome set at 96 beats per minute was used to standardise the rate of testing. The repetition number on which the first onset of pain occurred, or the first increase in pain for participants who had a constant background ache prior to testing, was recorded, up to a maximum of 25 repetitions.

The blinded assessor measured outcome at six, 12 and 52 weeks by way of worst pain VAS, KPS and FIQ, which have established reliability and validity in PFP [25,27-30].

### Statistical analysis

Statistical analysis was carried out using SPSS software (Version 15.0). The first stage of the analysis used univariate linear regression to investigate associations between each single potential prognostic factor and each outcome measure at six, 12 and 52 weeks. The baseline value of the outcome measure and the treatment group that participants were randomly assigned to within the randomised clinical trial were included as covariates in each analysis. Interactions between treatment group and each potential prognostic factor were evaluated to determine whether there existed any differences in prognostic variables between groups. For these interaction analyses, the flat insert group served as the reference group. This was done on the basis that flat inserts served as a control arm of the randomised clinical trial, as was borne out by the results thereof. A significance level of *p *≤ 0.1 rather than the conventional level of *p *< 0.05 was selected to ensure that the univariate analyses were sufficiently sensitive to identify potential prognostic factors for entry in the model. This is consistent with the work of others in prognostic studies of musculoskeletal conditions [10,31].

For the second stage, all potential prognostic factors that showed significant associations on univariate analyses were entered into a stepwise multivariate linear regression with backward elimination (*p *≤ 0.10) in order to identify a group of factors that were independently associated with poor outcome on each outcome measure at six, 12 and 52 weeks. This was conducted firstly without interactions, and then with significant interactions retained in the model. For the final multivariate models, the significance was set at 0.01 to minimise the results being adversely influenced by the likelihood of increased risk of Type I error associated with multiple analyses. The strength of the predictive ability of identified factors was determined with unstandardised regression coefficients (β) and their 95% confidence intervals, while the predictive power of each final model was given by calculation of the percentage of explained variance (adjusted R^2^).

## Results

### Prognostic indicators of outcome at six weeks

Univariate linear regression revealed that duration of knee pain, baseline scores of worst pain, FIQ and KPS, and number of pain-free step down repetitions were significantly associated with six-week KPS (additional file 1: Table S1). Of these, longer duration of knee pain (β-0.04, 95% confidence interval -0.07 to -0.01, *p *= 0.004) and lower baseline KPS (0.48, 0.31 to 0.65, *p *< 0.000) remained significantly associated with a lower six-week KPS in the multivariate model, which explained 40.1% of the total variance.

All potential prognostic indicators except BMI had significant univariate associations with six-week FIQ. Longer duration (-0.01, -0.02 to 0, *p *= 0.006) and lower baseline FIQ (0.56, 0.36 to 0.77, *p *< 0.000) remained significantly associated with lower six-week FIQ on multivariate analysis, which explained 38.6% of the total variance.

Age, duration, and baseline measures of worst pain, FIQ, KPS and pain-free step downs had significant univariate associations with six-week worst pain. The multivariate analysis showed that higher worst pain at baseline retained a significant association with a higher six-week worst pain (0.42, 0.16 to 0.69, *p *= 0.002; R^2 ^= 23%).

Significant univariate interactions between treatment group and age for all three outcome measures, as well as between treatment group and arch height for FIQ, did not significantly alter the predictor factors on outcome when included in the multivariate models.

### Prognostic indicators of outcome at 12 weeks

Baseline worst pain, KPS and pain-free step downs had significant univariate associations with 12-week KPS (additional file 1: Table S2). On multivariate analysis, lower baseline KPS retained a significant association with lower 12-week KPS (0.45, 0.27 to 0.64, *p *< 0.000). The interaction between treatment group and baseline FIQ was not significant in the final model, with its inclusion only increasing the percentage of explained total variance from 23.2% to 28%, with baseline KPS remaining a significant predictor (0.43, 0.18 to 0.68, *p *= 0.001).

Univariate analyses found duration and baseline scores for worst pain and KPS to be significantly associated with 12-week FIQ. In the multivariate model, which explained 22% of the total variance, lower baseline KPS remained significantly associated with lower FIQ at 12 weeks (0.12, 0.07 to 0.16, *p *< 0.000). The inclusion in the model of significant univariate interactions between treatment group and baseline FIQ, as well as treatment group and pain-free step downs, increased the explained variance to 34.7%; however KPS was no longer a significant predictor (0.08, 0.01 to 0.14, *p *= 0.019), nor were there any other significant associations.

On univariate analyses, age, duration, baseline scores of worst pain, FIQ and KPS, and number of pain-free step downs were significantly associated with 12-week worst pain. Multivariate analysis revealed no prognostic indicators of 12-week worst pain with or without the inclusion of the treatment group by duration interaction, which was significant on univariate analysis.

### Prognostic indicators of outcome at 52 weeks

Duration as well as worst pain and KPS at baseline had significant univariate associations with 52-week KPS (additional file 1: Table S3). Longer duration (-0.07, -0.1 to -0.03, *p *< 0.000) and lower baseline KPS (0.33, 0.13 to 0.53, *p *= 0.002) maintained this significant association in the multivariate model, which explained 29.5% of the total variance. When interactions between treatment group and duration as well as treatment group and FIQ were included, the association between KPS at baseline and 52 weeks remained significant (0.4, 0.17 to 0.63, *p *= 0.001), but the association between duration and 52-week KPS was not statistically significant (-0.11, -0.19 to -0.02, *p *= 0.013). The final model with interactions included explained 49.7% of the total variance.

Age, duration, baseline scores of worst pain, FIQ and KPS, and pain-free step downs were all significantly associated with 52-week FIQ on univariate analyses. Multivariate analysis revealed that long duration was significantly associated with low 52-week FIQ (-0.02, -0.03 to -0.01, *p *< 0.000), with the final model explaining 26.6% of the total variance. This did not change with the inclusion of the treatment group by age interaction.

On univariate analyses, duration and baseline scores of worst pain and KPS had significant associations with 52-week worst pain; however were not significant in the multivariate model (adjusted R^2 ^= 5.4%).

## Discussion

Multivariate regression analyses revealed that the two most consistent predictors of poor outcome in individuals with PFP over a 12-month period were a long duration of knee pain and a low baseline KPS. At six weeks, long pain duration was predictive of poor outcome on two of the three outcome measures (KPS and FIQ), while baseline scores of each measure that indicated greater severity of the condition not surprisingly predicted poor outcome on their respective measures. KPS was also found to be the sole prognostic indicator at 12 weeks when outcome was measured with the same questionnaire and the FIQ. Long pain duration was also predictive of poor outcome at 52 weeks when measured on the KPS and FIQ, while low KPS at baseline was predictive of poor 52-week outcome on the KPS.

Findings of our study are consistent with those of Mallen et al [11], whose systematic review of generic musculoskeletal pain found that longer pain duration and higher baseline disability were consistent prognostic indicators in musculoskeletal conditions. However, unlike Mallen et al [11] we did not find age to be predictive of outcome in our PFP cohort. This may be due to the difference in age ranges between studies. While our cohort was restricted to 18 to 40 years of age, the majority of studies in their systematic review included a greater age range of participants; frequently aged from 18 to at least 65 years [11]. It is also plausible that age is a condition-specific prognostic factor.

This study also confirms previous findings with respect to factors that are not predictive of outcome in PFP. Based on findings of ours and previous studies [15-17], gender does not appear to be a prognostic factor for PFP. The lack of association that we found between BMI and outcome is consistent with findings in both PFP [17] and general knee complaints [32], and implies that modifying or controlling BMI in those with PFP is unlikely to change their prognosis. One consideration is that, despite the inclusion of some individuals with a high BMI, overall this PFP group was not classified as overweight (Table 1). Hence, further studies may need to consider subgroup analysis based on BMI classification.

This study investigated the prognostic role of foot posture based on the long-hypothesised relationship between foot pronation, biomechanical function of the patellofemoral joint, and the genesis of PFP [20]. While this has been explored in a previous prognostic study [17], ours is the first to utilise a reliable measure [22]. A static measure of weight bearing arch height was selected as a representative measure of foot posture, on the basis that it is reliable and valid, well correlated with dynamic foot function, and is simple and efficient to obtain in a clinical setting [33,34]. Our finding that foot posture was not predictive of outcome in PFP is consistent with previous studies that failed to identify any other lower limb biomechanical measures as prognostic factors [15-17], although further research to confirm this for biomechanical measures other than foot posture is warranted.

While this study has identified pain duration and KPS as prognostic indicators, there may be other factors of a physical or psychological nature that are stronger predictors of outcome for PFP. Our findings provide guidelines as to physical factors worthy of inclusion in and exclusion from future prognostic studies. Psychological prognostic factors identified for other chronic musculoskeletal conditions such as anxiety and depression [11], and coping strategies such as catastrophising [35], require investigation in a PFP cohort.

Given that persistent knee pain has been implicated as a precursor to osteoarthritis [36,37], the findings of this study are of substantial individual and public health importance. Our results suggest that, in order to optimise prognosis of individuals with PFP, it is best to attempt to prevent chronicity and minimise the severity of the condition. This may be achieved through education regarding the importance of early intervention to minimise duration. Intervention should aim to improve scores on the KPS, worst pain VAS and FIQ as quickly as possible, utilising modalities such as foot orthoses and multimodal physiotherapy that have the best evidence for efficacy in PFP [19,24], as well as advice to avoid pain-provoking activities while maintaining an otherwise physically active lifestyle. Future clinical trials should also investigate the ability of other pain-relieving modalities to reduce pain more rapidly, such as electrotherapy, transcutaneous electrical nerve stimulation, analgesics, anti-inflammatory medication and acupuncture. Furthermore, KPS, worst pain VAS and FIQ scores that constitute low versus high severity need to be determined to facilitate more precise clinical application.

The inclusion of significant interactions between treatment group and potential prognostic variables in the multivariate model largely did not affect conclusions drawn from the final model, although there was generally an increase in the percentage of total variability explained by the model. This indicates that the prognostic factors identified for the entire cohort were not modified by any of the interventions.

Compared to previous prognostic studies, our study sought to control a number of factors that could compromise the external validity of its findings. Firstly, we have attempted to control for possible differential treatment effects and analyse outcome independent of intervention received. Because the previous four studies assigned the same standardised treatment program to all participants, their results look at the predictive ability of particular variables when treated with a standardised program [14-17]. In contrast, participants in the current study were randomly assigned to one of four different interventions, and treatment effects were accounted for in analyses through inclusion of treatment as a covariate. Secondly, we utilised outcome measures that have been previously established as reliable and valid for PFP. Thirdly, our methodology addresses a shortfall of previous musculoskeletal prognostic studies identified by Mallen et al [11], in that the measures investigated for potential predictive ability and those used to measure outcome have high clinical utility and are easily applied in a clinical setting.

## Conclusions

This study has identified simple, clinically appropriate and reliable condition-specific measures that are predictive of poor outcome in individuals with PFP. Patients presenting with PFP of long duration and a low KPS should be flagged as potentially having a poorer outcome over time, irrespective of their age, gender and morphometry. These findings suggest that attempting to prevent PFP chronicity by using efficacious interventions early after the onset of symptoms may optimise prognosis for this patient group, which may have important implications for prevention of potential sequelae of long term pain and disability.

## Competing interests

The authors declare that they have no competing interests.

## Authors' contributions

NC, RD and BV designed the study. NC performed statistical analyses with input from RD, while NC, RD and BV interpreted statistical findings. NC, KC and BV drafted the manuscript. All authors read and approved the final manuscript.

## Pre-publication history

The pre-publication history for this paper can be accessed here:

http://www.biomedcentral.com/1471-2474/11/11/prepub

## Supplementary Material

Additional file 1 **Results of six, 12 and 52-week prognostic analyses**. Table S1. Prognostic indicators of outcome at six weeks (n = 164^) (adjusted for treatment group). Table S2. Prognostic indicators of outcome at 12 weeks (n = 161^) (adjusted for treatment group). Table S3. Prognostic indicators of outcome at 52 weeks (n = 170, 145, 171, respectively^) (adjusted for treatment group). Click here for file

## References

[B1] Witvrouw E Lysens R Bellemans J Cambier D Vanderstraeten G Intrinsic risk factors for the development of anterior knee pain in an athletic population. A two-year prospective study Am J Sports Med 2000 28 4 480 489 10921638 10.1177/03635465000280040701

[B2] Hetsroni I Finestone A Milgrom C Sira DB Nyska M Radeva-Petrova D Ayalon M A prospective biomechanical study of the association between foot pronation and the incidence of anterior knee pain among military recruits J Bone Joint Surg Br 2006 88 7 905 908 10.1302/0301-620X.88B7.17826 16798993

[B3] Milgrom C Finestone A Eldad A Shlamkovitch N Patellofemoral pain caused by overactivity. A prospective study of risk factors in infantry recruits J Bone Joint Surg Am 1991 73 7 1041 1043 1874766

[B4] Schwellnus MP Jordaan G Noakes TD Prevention of common overuse injuries by the use of shock absorbing insoles. A prospective study Am J Sports Med 1990 18 6 636 641 10.1177/036354659001800614 2285093

[B5] Van Tiggelen D Cowan S Coorevits P Duvigneaud N Witvrouw E Delayed vastus medialis obliquus to vastus lateralis onset timing contributes to the development of patellofemoral pain in previously healthy men American Journal of Sports Medicine 2009 37 6 1099 1105 10.1177/0363546508331135 19282508

[B6] Wills AK Ramasamy A Ewins DJ Etherington J The incidence and occupational outcome of overuse anterior knee pain during army recruit training J R Army Med Corps 2004 150 4 264 269 15732414 10.1136/jramc-150-04-07

[B7] Australian Acute Musculoskeletal Pain Guidelines Group Evidence-based management of acute musculoskeletal pain 2003 Brisbane: Australian Academic Press Pty. Ltd

[B8] Nimon G Murray D Sandow M Goodfellow J Natural history of anterior knee pain: a 14- to 20-year follow-up of nonoperative management J Pediatr Orthop 1998 18 1 118 122 10.1097/00004694-199801000-00021 9449112

[B9] Milgrom C Finestone A Shlamkovitch N Giladi M Radin E Anterior knee pain caused by overactivity: a long term prospective followup Clin Orthop Relat Res 1996 331 256 260 10.1097/00003086-199610000-00036 8895647

[B10] Thomas E Windt DA van der Hay EM Smidt N Dziedzic K Bouter LM Croft PR Two pragmatic trials of treatment for shoulder disorders in primary care: generalisability, course, and prognostic indicators Ann Rheum Dis 2005 64 7 1056 1061 10.1136/ard.2004.029959 15640264 PMC1755568

[B11] Mallen CD Peat G Thomas E Dunn KM Croft PR Prognostic factors for musculoskeletal pain in primary care: a systematic review Br J Gen Pract 2007 57 541 655 661 17688762 PMC2099673

[B12] Jewell DV Guide to Evidence-Based Physical Therapy Practice 2008 Sudbury, Massachusetts: Jones and Bartlett Publishers, Inc

[B13] Straus SE Richardson WS Glasziou P Haynes RB Evidence-Based Medicine. How to Practice and Teach EBM 2005 Third Edinburgh: Elsevier

[B14] Selfe J Harper L Pedersen I Breen-Turner J Waring J Stevens D Cold legs: a potential indicator of negative outcome in the rehabilitation of patients with patellofemoral pain syndrome Knee 2003 10 2 139 143 10.1016/S0968-0160(02)00085-6 12787996

[B15] Witvrouw E Lysens R Bellemans J Cambier D Cools A Danneels L Bourgois J Which factors predict outcome in the treatment program of anterior knee pain? Scand J Med Sci Sports 2002 12 1 40 46 10.1034/j.1600-0838.2002.120108.x 11985765

[B16] Natri A Kannus P Jarvinen M Which factors predict the long-term outcome in chronic patellofemoral pain syndrome? A 7-yr prospective follow-up study Med Sci Sports Exerc 1998 30 11 1572 1577 10.1097/00005768-199811000-00003 9813868

[B17] Kannus P Niittymaki S Which factors predict outcome in the nonoperative treatment of patellofemoral pain syndrome? A prospective follow-up study Med Sci Sports Exerc 1994 26 3 289 296 8183092

[B18] Boling M Padua D Marshall S Guskiewicz K Pyne S Beutler A Gender differences in the incidence and prevalence of patellofemoral pain syndrome Scandinavian Journal of Medicine & Science in Sports 2009 9999 9999 1 6 10.1111/j.1600-0838.2009.00996.x PMC2895959 19765240

[B19] Collins N Crossley K Beller E Darnell R McPoil T Vicenzino B Foot orthoses and physiotherapy in the treatment of patellofemoral pain syndrome: randomised clinical trial BMJ 2008 337 a1735 10.1136/bmj.a1310 18952682 PMC2572211

[B20] Tiberio D The effect of excessive subtalar joint pronation on patellofemoral mechanics: A theoretical model Journal of Orthopaedic and Sports Physical Therapy 1987 9 4 160 165 18797010 10.2519/jospt.1987.9.4.160

[B21] van Dijk CN Tempel WM van der Patellofemoral pain syndrome Bmj 2008 337 a1948 10.1136/bmj.a1948 18952683

[B22] McPoil TG Vicenzino B Cornwall MW Collins N Warren M Reliability and normative values for the foot mobility magnitude: a composite measure of vertical and medial-lateral mobility of the midfoot Journal of Foot and Ankle Research 2009 2 6 10.1186/1757-1146-2-6 19267907 PMC2656480

[B23] Vicenzino B Collins N Crossley K Beller E Darnell R McPoil T Foot orthoses and physiotherapy in the treatment of patellofemoral pain syndrome: a randomised clinical trial BMC Musculoskelet Disord 2008 9 1 27 10.1186/1471-2474-9-27 18304317 PMC2279129

[B24] Crossley K Bennell K Green S Cowan S McConnell J Physical therapy for patellofemoral pain: a randomized, double-blinded, placebo-controlled trial Am J Sports Med 2002 30 6 857 865 12435653 10.1177/03635465020300061701

[B25] Harrison E Quinney H Magee D Sheppard MS McQuarrie A Analysis of outcome measures used in the study of patellofemoral pain syndrome Physiother Can 1995 47 4 264 272 10153395

[B26] Kujala UM Jaakkola LH Koskinen SK Taimela S Hurme M Nelimarkka O Scoring of patellofemoral disorders Arthroscopy 1993 9 2 159 163 8461073 10.1016/s0749-8063(05)80366-4

[B27] Chesworth BM Culham EG Tata GE Peat M Validation of outcome measures in patients with patellofemoral syndrome J Orthop Sports Phys Ther 1989 10 8 302 308 18796951 10.2519/jospt.1989.10.8.302

[B28] Bennell K Bartrim S Crossley K Green S Outcome measures in patellofemoral pain syndrome: test-retest reliability and inter-relationships Physical Therapy in Sport 2000 1 32 41 10.1054/ptsp.2000.0009

[B29] Crossley KM Bennell KL Cowan SM Green S Analysis of outcome measures for persons with patellofemoral pain: which are reliable and valid? Arch Phys Med Rehabil 2004 85 5 815 822 10.1016/S0003-9993(03)00613-0 15129407

[B30] Watson CJ Propps M Ratner J Zeigler DL Horton P Smith SS Reliability and responsiveness of the lower extremity functional scale and the anterior knee pain scale in patients with anterior knee pain J Orthop Sports Phys Ther 2005 35 3 136 146 15839307 10.2519/jospt.2005.35.3.136

[B31] Smidt N Lewis M DA VDW Hay EM Bouter LM Croft P Lateral epicondylitis in general practice: course and prognostic indicators of outcome J Rheumatol 2006 33 10 2053 2059 16881095

[B32] Waal JM van der Bot SD Terwee CB Windt DA van der Scholten RJ Bouter LM Dekker J Course and prognosis of knee complaints in general practice Arthritis Rheum 2005 53 6 920 930 10.1002/art.21581 16342106

[B33] Franettovich MM McPoil TG Russell T Skardoon G Vicenzino B The ability to predict dynamic foot posture from static measurements J Am Podiatr Med Assoc 2007 97 2 115 120 17369317 10.7547/0970115

[B34] McPoil TG Cornwall MW Vicenzino B Teyhen DS Molloy JM Christie DS Collins N Effect of using truncated versus total foot length to calculate the arch height ratio Foot 2008 18 4 220 227 10.1016/j.foot.2008.06.002 20307441

[B35] Walton D Pretty J Macdermid J Teasell R Risk factors for persistent problems following whiplash injury: results of a systematic review and meta-analysis Journal of Orthopaedic and Sports Physical Therapy 2009 39 5 334 350 19411766 10.2519/jospt.2009.2765

[B36] Thorstensson CA Petersson IF Jacobsson LT Boegard TL Roos EM Reduced functional performance in the lower extremity predicted radiographic knee osteoarthritis five years later Ann Rheum Dis 2004 63 4 402 407 10.1136/ard.2003.007583 15020334 PMC1754965

[B37] Thorstensson CA Andersson ML Jonsson H Saxne T Petersson IF The natural course of knee osteoarthritis in middle-aged individuals with knee pain - A 12 year follow-up using clinical and radiographic criteria Ann Rheum Dis 2008 in press 19054828 10.1136/ard.2008.095158

