# Supplementary material for: Predictors of short and long term outcome in patellofemoral pain syndrome: a prospective longitudinal study
Source: BMC Musculoskelet Disord. 2010 Jan 19;11:11. doi: 10.1186/1471-2474-11-11 (PMC2823664; doi:10.1186/1471-2474-11-11)
Supplement: Additional file 1 — Results of six, 12 and 52-week prognostic analyses. Table S1. Prognostic indicators of outcome at six weeks (n = 164^) (adjusted for treatment group). Table S2. Prognostic indicators of outcome at 12 weeks (n = 161^) (adjusted for treatment group). Table S3. Prognostic indicators of outcome at 52 weeks (n = 170, 145, 171, respectively^) (adjusted for treatment group). [file 1471-2474-11-11-S1.DOC]

Table 1. Prognostic indicators of outcome at six weeks (n = 164^) (adjusted for treatment group).

|  | **Univariate analysis** | |  | **Multivariate analysis** | |  | **Multivariate analysis**  **(with interactions)** | |
| --- | --- | --- | --- | --- | --- | --- | --- | --- |
| **Variables** | **β (95% CI)** | ***p*** |  | **β (95% CI)** | ***p*** |  | **β (95% CI)** | ***p*** |
| **WORST PAIN VISUAL ANALOGUE SCALE** | |  |  |  |  |  |  |  |
| Intercept |  |  |  | 23.68 (6.15 to 41.2) | 0.009 |  | 23.68 (6.15 to 41.2) | 0.009 |
| ***Treatment group (ref: Flat inserts):*** | |  |  |  |  |  |  |  |
| FO+PT | -15.91 (-25.72 to -6.09) | 0.002 |  | -24.73 (-36.86 to -12.6) | 0.000 |  | -24.73 (-36.86 to -12.6) | 0.000 |
| PT | -13.62 (-23.49 to -3.75) | 0.007 |  | -16.48 (-28.45 to -4.5) | 0.008 |  | -16.48 (-28.45 to -4.5) | 0.008 |
| FO | -7.24 (-17.11 to 2.63) | 0.149 |  | -15.22 (-27.29 to -3.16) | 0.014 |  | -15.22 (-27.29 to -3.16) | 0.014 |
| ***Prognostic indicators:*** |  |  |  |  |  |  |  |  |
| Age at baseline | ***-1.33 (-2.51 to -0.14)*** | ***0.029*** |  |  |  |  |  |  |
| *Interaction FO+PT x Age* | ***1.95 (0.27 to 3.62)*** | ***0.023*** |  |  |  |  |  |  |
| *Interaction PT x Age* | 0.33 (-1.34 to 2) | 0.700 |  |  |  |  |  |  |
| *Interaction FO x Age* | 0.3 (-1.41 to 2.01) | 0.731 |  |  |  |  |  |  |
| Gender | 5.87 (-1.16 to 12.9) | 0.101 |  |  |  |  |  |  |
| Body Mass Index | -0.52 (-1.22 to 0.19) | 0.151 |  |  |  |  |  |  |
| Duration of knee pain | ***0.06 (0 to 0.12)*** | ***0.059*** |  | 0.09 (0.02 to 0.16) | 0.015 |  | 0.09 (0.02 to 0.16) | 0.015 |
| VAS worst (baseline) | ***0.41 (0.2 to 0.63)*** | ***0.000*** |  | **0.42 (0.16 to 0.69)** | **0.002** |  | **0.42 (0.16 to 0.69)** | **0.002** |
| FIQ (baseline) | ***-2.81 (-4.43 to -1.19)*** | ***0.001*** |  |  |  |  |  |  |
| KPS (baseline) | ***-0.56 (-0.91 to -0.22)*** | ***0.002*** |  |  |  |  |  |  |
| Arch height (study side) | -0.41 (-1.14 to 0.33) | 0.274 |  |  |  |  |  |  |
| Pain-free step downs (baseline) | ***-0.82 (-1.51 to -0.13)*** | ***0.020*** |  | -0.58 (-1.45 to 0.29) | 0.189 |  | -0.58 (-1.45 to 0.29) | 0.189 |
| **Adjusted R^2^** |  |  |  | **23%** |  |  | **23%** |  |
| **KUJALA PATELLOFEMORAL SCORE** | |  |  |  |  |  |  |  |
| Intercept |  |  |  | 40.99 (27.74 to 54.24) | 0.000 |  | 40.99 (27.74 to 54.24) | 0.000 |
| ***Treatment group (ref: Flat inserts):*** | |  |  |  |  |  |  |  |
| FO+PT | 8.4 (3.81 to 13) | 0.000 |  | 11.03 (6.21 to 15.85) | 0.000 |  | 11.03 (6.21 to 15.85) | 0.000 |
| PT | 8.34 (3.72 to 12.95) | 0.000 |  | 10.69 (5.88 to 15.5) | 0.000 |  | 10.69 (5.88 to 15.5) | 0.000 |
| FO | 4.29 (-0.33 to 8.91) | 0.068 |  | 7.11 (2.28 to 11.95) | 0.004 |  | 7.11 (2.28 to 11.95) | 0.004 |
| ***Prognostic indicators:*** |  |  |  |  |  |  |  |  |
| Age at baseline | 0.37 (-0.19 to 0.93) | 0.190 |  |  |  |  |  |  |
| *Interaction FO+PT x Age* | ***-0.89 (-1.68 to -0.1)*** | ***0.027*** |  |  |  |  |  |  |
| *Interaction PT x Age* | -0.27 (-1.05 to 0.52) | 0.500 |  |  |  |  |  |  |
| *Interaction FO x Age* | 0.27 (-0.54 to 1.07) | 0.512 |  |  |  |  |  |  |
| Gender | -1.44 (-4.75 to 1.86) | 0.390 |  |  |  |  |  |  |
| Body Mass Index | 0.08 (-0.25 to 0.41) | 0.635 |  |  |  |  |  |  |
| Duration of knee pain | ***-0.06 (-0.09 to -0.03)*** | ***0.000*** |  | **-0.04 (-0.07 to -0.01)** | **0.004** |  | **-0.04 (-0.07 to -0.01)** | **0.004** |
| VAS worst (baseline) | ***-0.19 (-0.29 to -0.09)*** | ***0.000*** |  |  |  |  |  |  |
| FIQ (baseline) | ***2.23 (1.52 to 2.93)*** | ***0.000*** |  |  |  |  |  |  |
| KPS (baseline) | ***0.54 (0.39 to 0.68)*** | ***0.000*** |  | **0.48 (0.31 to 0.65)** | **0.000** |  | **0.48 (0.31 to 0.65)** | **0.000** |
| Arch height (study side) | 0.15 (-0.19 to 0.48) | 0.393 |  |  |  |  |  |  |
| Pain-free step downs (baseline) | ***0.36 (0.04 to 0.69)*** | ***0.027*** |  |  |  |  |  |  |
| **Adjusted R^2^** |  |  |  | **40.1%** |  |  | **40.1%** |  |
| **FUNCTIONAL INDEX QUESTIONNAIRE** | |  |  |  |  |  |  |  |
| Intercept |  |  |  | 5.91 (-1.36 to 13.18) | 0.110 |  | 5.91 (-1.36 to 13.18) | 0.110 |
| ***Treatment group (ref: Flat inserts):*** | |  |  |  |  |  |  |  |
| FO+PT | 1.73 (0.63 to 2.82) | 0.002 |  | 2.71 (1.47 to 3.96) | 0.000 |  | 2.71 (1.47 to 3.96) | 0.000 |
| PT | 1.63 (0.53 to 2.73) | 0.004 |  | 2.3 (1.05 to 3.56) | 0.000 |  | 2.3 (1.05 to 3.56) | 0.000 |
| FO | 0.7 (-0.4 to 1.8) | 0.210 |  | 1 (-0.26 to 2.26) | 0.120 |  | 1 (-0.26 to 2.26) | 0.120 |
| ***Prognostic indicators:*** |  |  |  |  |  |  |  |  |
| Age at baseline | ***0.13 (-0.01 to 0.26)*** | ***0.064*** |  |  |  |  |  |  |
| *Interaction FO+PT x Age* | ***-0.23 (-0.42 to -0.05)*** | ***0.015*** |  |  |  |  |  |  |
| *Interaction PT x Age* | -0.08 (-0.27 to 0.11) | 0.402 |  |  |  |  |  |  |
| *Interaction FO x Age* | 0.02 (-0.17 to 0.21) | 0.814 |  |  |  |  |  |  |
| Gender | ***-0.98 (-1.76 to -0.21)*** | ***0.013*** |  | -1.32 (-2.37 to -0.27) | 0.014 |  | -1.32 (-2.37 to -0.27) | 0.014 |
| Body Mass Index | -0.03 (-0.11 to 0.05) | 0.434 |  |  |  |  |  |  |
| Duration of knee pain | ***-0.01 (-0.02 to -0.01)*** | ***0.001*** |  | **-0.01 (-0.02 to 0)** | **0.006** |  | **-0.01 (-0.02 to 0)** | **0.006** |
| VAS worst (baseline) | ***-0.04 (-0.06 to -0.01)*** | ***0.004*** |  |  |  |  |  |  |
| FIQ (baseline) | ***0.53 (0.36 to 0.7)*** | ***0.000*** |  | **0.56 (0.36 to 0.77)** | **0.000** |  | **0.56 (0.36 to 0.77)** | **0.000** |
| KPS (baseline) | ***0.1 (0.06 to 0.13)*** | ***0.000*** |  |  |  |  |  |  |
| Arch height (study side) | ***0.25 (0.06 to 0.45)*** | ***0.012*** |  | 0.03 (-0.06 to 0.11) | 0.510 |  | 0.03 (-0.06 to 0.11) | 0.510 |
| *Interaction FO+PT x Arch height* | ***-0.33 (-0.57 to -0.08)*** | ***0.011*** |  |  |  |  |  |  |
| *Interaction PT x Arch height* | ***-0.25 (-0.51 to 0.02)*** | ***0.066*** |  |  |  |  |  |  |
| *Interaction FO x Arch height* | -0.2 (-0.45 to 0.06) | 0.132 |  |  |  |  |  |  |
| Pain-free step downs (baseline) | ***0.08 (0 to 0.15)*** | ***0.058*** |  |  |  |  |  |  |
| **Adjusted R^2^** |  |  |  | **38.6%** |  |  | **38.6%** |  |

^ n less than 179 due to missing data; FO+PT foot orthoses plus physiotherapy; PT physiotherapy; FO foot orthoses

***Included in multivariate analysis (significant at p = 0.1)***. **Significant at *p* = 0.01**

Table 2. Prognostic indicators of outcome at 12 weeks (n = 161^) (adjusted for treatment group).

|  | **Univariate analysis** | |  | **Multivariate analysis** | |  | **Multivariate analysis**  **(with interactions)** | |
| --- | --- | --- | --- | --- | --- | --- | --- | --- |
| **Variables** | **β (95% CI)** | ***p*** |  | **β (95% CI)** | ***p*** |  | **β (95% CI)** | ***p*** |
| **WORST PAIN VISUAL ANALOGUE SCALE** | |  |  |  |  |  |  |  |
| Intercept |  |  |  | 38.51 (7.82 to 69.19) | 0.014 |  | 26.11 (-5.08 to 57.3) | 0.100 |
| ***Treatment group (ref: Flat inserts):*** | |  |  |  |  |  |  |  |
| FO+PT | -4.17 (-14.59 to 6.25) | 0.430 |  | -17.15 (-30.36 to -3.94) | 0.012 |  | -0.43 (-18.22 to 17.36) | 0.962 |
| PT | -5.32 (-15.67 to 5.04) | 0.312 |  | -12.59 (-25.65 to 0.48) | 0.059 |  | -9.05 (-26.99 to 8.9) | 0.319 |
| FO | 0.33 (-9.97 to 10.63) | 0.949 |  | -6.82 (-19.79 to 6.15) | 0.299 |  | 0.65 (-17.3 to 18.6) | 0.943 |
| ***Prognostic indicators:*** |  |  |  |  |  |  |  |  |
| Age at baseline | ***-0.6 (-1.23 to 0.04)*** | ***0.064*** |  | -0.52 (-1.31 to 0.26) | 0.190 |  | -0.38 (-1.16 to 0.39) | 0.328 |
| Gender | -0.25 (-7.68 to 7.18) | 0.948 |  |  |  |  |  |  |
| Body Mass Index | 0.01 (-0.71 to 0.73) | 0.974 |  |  |  |  |  |  |
| Duration of knee pain | ***0.17 (0.01 to 0.33)*** | ***0.038*** |  |  |  |  | 0.23 (0.01 to 0.46) | 0.045 |
| *Interaction FO+PT x Duration* | ***-0.26 (-0.46 to -0.07)*** | ***0.009*** |  |  |  |  | -0.34 (-0.59 to -0.08) | 0.011 |
| *Interaction PT x Duration* | -0.07 (-0.27 to 1.3) | 0.481 |  |  |  |  | -0.12 (-0.38 to 0.15) | 0.394 |
| *Interaction FO x Duration* | -0.13 (-0.34 to 0.08) | 0.220 |  |  |  |  | -0.17 (-0.45 to 0.11) | 0.235 |
| VAS worst (baseline) | ***0.47 (0.25 to 0.69)*** | ***0.000*** |  | 0.34 (0.06 to 0.62) | 0.020 |  | 0.31 (0.04 to 0.59) | 0.027 |
| FIQ (baseline) | ***-3.37 (-5.03 to -1.71)*** | ***0.000*** |  |  |  |  |  |  |
| KPS (baseline) | ***-0.59 (-0.97 to -0.22)*** | ***0.002*** |  |  |  |  |  |  |
| Arch height (study side) | -0.12 (-0.84 to 0.6) | 0.737 |  |  |  |  |  |  |
| Pain-free step downs (baseline) | ***-0.92 (-1.63 to -0.21)*** | ***0.011*** |  | -0.79 (-1.72 to 0.13) | 0.092 |  | -0.91 (-1.82 to 0) | 0.049 |
| **Adjusted R^2^** |  |  |  | **13.1%** |  |  | **19.1%** |  |
| **KUJALA PATELLOFEMORAL SCORE** | |  |  |  |  |  |  |  |
| Intercept |  |  |  | 47.28 (32.87 to 61.69) | 0.000 |  | 46.59 (20.36 to 72.82) | 0.001 |
| ***Treatment group (ref: Flat inserts):*** | |  |  |  |  |  |  |  |
| FO+PT | 5.33 (0.46 to 10.2) | 0.032 |  | 8.34 (2.81 to 13.86) | 0.004 |  | 19.31 (-9.05 to 47.67) | 0.180 |
| PT | 3.53 (-1.31 to 8.37) | 0.151 |  | 5.31 (-0.24 to 10.85) | 0.061 |  | 9.72 (-17.30 to 36.73) | 0.477 |
| FO | 0.08 (-4.73 to 4.9) | 0.973 |  | 1.92 (-3.56 to 7.39) | 0.489 |  | -22.09 (-50.90 to 6.71) | 0.131 |
| ***Prognostic indicators:*** |  |  |  |  |  |  |  |  |
| Age at baseline | 0.07 (-0.51 to 0.65) | 0.810 |  |  |  |  |  |  |
| *Interaction FO+PT x Age* | -0.68 (-1.51 to 0.15) | 0.109 |  |  |  |  |  |  |
| *Interaction PT x Age* | -0.1 (-0.91 to 0.72) | 0.815 |  |  |  |  |  |  |
| *Interaction FO x Age* | ***0.7 (-0.13 to 1.53)*** | ***0.100*** |  |  |  |  |  |  |
| Gender | 0.51 (-2.96 to 3.99) | 0.771 |  |  |  |  |  |  |
| Body Mass Index | -0.1 (-0.43 to 0.24) | 0.569 |  |  |  |  |  |  |
| Duration of knee pain | -0.06 (-0.13 to 0.02) | 0.134 |  |  |  |  |  |  |
| *Interaction FO+PT x Duration* | 0.07 (-0.02 to 0.16) | 0.101 |  |  |  |  |  |  |
| *Interaction PT x Duration* | -0.05 (-0.14 to 0.04) | 0.257 |  |  |  |  |  |  |
| *Interaction FO x Duration* | 0.02 (-0.07 to 0.12) | 0.627 |  |  |  |  |  |  |
| VAS worst (baseline) | ***-0.11 (-0.22 to 0)*** | ***0.050*** |  |  |  |  |  |  |
| FIQ (baseline) | 1.18 (-0.61 to 2.98) | 0.195 |  |  |  |  | 0.26 (-2.03 to 2.56) | 0.820 |
| *Interaction FO+PT x FIQ* | 0.46 (-1.87 to 2.79) | 0.698 |  |  |  |  | -1.13 (-3.96 to 1.70) | 0.430 |
| *Interaction PT x FIQ* | 0.29 (-1.88 to 2.45) | 0.795 |  |  |  |  | -0.45 (-3.10 to 2.20) | 0.736 |
| *Interaction FO x FIQ* | ***2.81 (0.41 to 5.21)*** | ***0.022*** |  |  |  |  | 2.38 (-0.45 to 5.21) | 0.099 |
| KPS (baseline) | ***0.48 (0.32 to 0.64)*** | ***0.000*** |  | **0.45 (0.27 to 0.64)** | **0.000** |  | **0.43 (0.18 to 0.68)** | **0.001** |
| Arch height (study side) | 0.06 (-0.27 to 0.39) | 0.727 |  |  |  |  |  |  |
| Pain-free step downs (baseline) | ***0.4 (0.07 to 0.73)*** | ***0.019*** |  |  |  |  |  |  |
| **Adjusted R^2^** |  |  |  | **23.2%** |  |  | **28.0%** |  |
| **FUNCTIONAL INDEX QUESTIONNAIRE** | |  |  |  |  |  |  |  |
| Intercept |  |  |  | 3.43 (-0.27 to 7.13) | 0.069 |  | 4.89 (-1.57 to 11.34) | 0.136 |
| ***Treatment group (ref: Flat inserts):*** | |  |  |  |  |  |  |  |
| FO+PT | 1.36 (0.22 to 2.51) | 0.019 |  | 1.92 (0.5 to 3.34) | 0.009 |  | 0.75 (-6.2 to 7.69) | 0.831 |
| PT | 1.11 (-0.03 to 2.24) | 0.056 |  | 1.6 (0.17 to 3.02) | 0.028 |  | 3.39 (-3.29 to 10.07) | 0.316 |
| FO | 0.15 (-0.98 to 1.27) | 0.798 |  | 0.52 (-0.88 to 1.93) | 0.461 |  | -7.35 (-14.58 to -0.12) | 0.046 |
| ***Prognostic indicators:*** |  |  |  |  |  |  |  |  |
| Age at baseline | 0.04 (-0.1 to 0.18) | 0.585 |  |  |  |  |  |  |
| *Interaction FO+PT x Age* | -0.14 (-0.34 to 0.06) | 0.162 |  |  |  |  |  |  |
| *Interaction PT x Age* | -0.05 (-0.25 to 0.14) | 0.577 |  |  |  |  |  |  |
| *Interaction FO x Age* | 0.12 (-0.08 to 0.32) | 0.237 |  |  |  |  |  |  |
| Gender | -0.05 (-0.86 to 0.77) | 0.914 |  |  |  |  |  |  |
| Body Mass Index | -0.04 (-0.12 to 0.04) | 0.346 |  |  |  |  |  |  |
| Duration of knee pain | ***-0.01 (-0.02 to 0)*** | ***0.004*** |  |  |  |  |  |  |
| VAS worst (baseline) | ***-0.04 (-0.06 to -0.01)*** | ***0.003*** |  |  |  |  |  |  |
| FIQ (baseline) | 0.21 (-0.19 to 0.62) | 0.297 |  |  |  |  | 0.21 (-0.35 to 0.77) | 0.449 |
| *Interaction FO+PT x FIQ* | 0.35 (-0.18 to 0.87) | 0.196 |  |  |  |  | 0.04 (-0.65 to 0.74) | 0.900 |
| *Interaction PT x FIQ* | 0.12 (-0.37 to 0.6) | 0.642 |  |  |  |  | -0.35 (-1.02 to 0.31) | 0.295 |
| *Interaction FO x FIQ* | ***0.87 (0.33 to 1.41)*** | ***0.002*** |  |  |  |  | 0.74 (0.01 to 1.46) | 0.047 |
| KPS (baseline) | ***0.1 (0.06 to 0.14)*** | ***0.000*** |  | **0.12 (0.07 to 0.16)** | **0.000** |  | 0.08 (0.01 to 0.14) | 0.019 |
| Arch height (study side) | -0.01 (-0.09 to 0.08) | 0.842 |  |  |  |  |  |  |
| Pain-free step downs (baseline) | -0.08 (-0.24 to 0.08) | 0.347 |  |  |  |  | -0.22 (-0.54 to 0.1) | 0.178 |
| *Interaction FO+PT x Step downs* | ***0.2 (-0.03 to 0.43)*** | ***0.092*** |  |  |  |  | 0.28 (-0.08 to 0.65) | 0.127 |
| *Interaction PT x Step downs* | ***0.29 (0.06 to 0.52)*** | ***0.016*** |  |  |  |  | 0.49 (0.1 to 0.88) | 0.014 |
| *Interaction FO x Step downs* | ***0.21 (0.01 to 0.42)*** | ***0.041*** |  |  |  |  | 0.19 (-0.17 to 0.54) | 0.300 |
| **Adjusted R^2^** |  |  |  | **22%** |  |  | **34.7%** |  |

^ n less than 179 due to missing data; FO+PT foot orthoses plus physiotherapy; PT physiotherapy; FO foot orthoses

***Included in multivariate analysis (significant at p = 0.1)***. **Significant at *p* = 0.01**

Table 3. Prognostic indicators of outcome at 52 weeks (n = 170, 145, 171, respectively^) (adjusted for treatment group).

|  | **Univariate analysis** | |  | **Multivariate analysis** | |  | **Multivariate analysis**  **(with interactions)** | |
| --- | --- | --- | --- | --- | --- | --- | --- | --- |
| **Variables** | **β (95% CI)** | ***p*** |  | **β (95% CI)** | ***p*** |  | **β (95% CI)** | ***p*** |
| **WORST PAIN VISUAL ANALOGUE SCALE** | |  |  |  |  |  |  |  |
| Intercept |  |  |  | 55.45 (10.42 to 100.49) | 0.016 |  |  |  |
| ***Treatment group (ref: Flat inserts):*** | |  |  |  |  |  |  |  |
| FO+PT | -4.57 (-15 to 5.86) | 0.388 |  | -5.11 (-17.83 to 7.6) | 0.427 |  |  |  |
| PT | -2.47 (-12.96 to 8.02) | 0.642 |  | -5.12 (-17.78 to 7.54) | 0.424 |  |  |  |
| FO | 2.55 (-7.82 to 12.92) | 0.628 |  | -0.23 (-12.63 to 12.18) | 0.971 |  |  |  |
| ***Prognostic indicators:*** |  |  |  |  |  |  |  |  |
| Age at baseline | -0.23 (-0.89 to 0.42) | 0.485 |  |  |  |  |  |  |
| Gender | -2.55 (-10.01 to 4.92) | 0.501 |  |  |  |  |  |  |
| Body Mass Index | 0.04 (-0.69 to 0.77) | 0.914 |  |  |  |  |  |  |
| Duration of knee pain | ***0.07 (0 to 0.14)*** | ***0.039*** |  |  |  |  |  |  |
| VAS worst (baseline) | ***0.32 (0.1 to 0.55)*** | ***0.006*** |  | 0.14 (-0.16 to 0.43) | 0.357 |  |  |  |
| FIQ (baseline) | -1.22 (-2.97 to 0.54) | 0.173 |  |  |  |  |  |  |
| KPS (baseline) | ***-0.76 (-1.12 to -0.39)*** | ***0.000*** |  | -0.55 (-1.02 to -0.08) | 0.023 |  |  |  |
| Arch height (study side) | 0.51 (-0.2 to 1.23) | 0.157 |  |  |  |  |  |  |
| Pain-free step downs (baseline) | -0.45 (-1.16 to 0.27) | 0.218 |  |  |  |  |  |  |
| **Adjusted R^2^** |  |  |  | **5.4%** |  |  |  |  |
| **KUJALA PATELLOFEMORAL SCORE** | |  |  |  |  |  |  |  |
| Intercept |  |  |  | 66.13 (50.68 to 81.78) | 0.000 |  | 83.13 (58.78 to 107.47) | 0.000 |
| ***Treatment group (ref: Flat inserts):*** | |  |  |  |  |  |  |  |
| FO+PT | 5.51 (0.51 to 10.51) | 0.031 |  | 6.15 (0.43 to 11.88) | 0.035 |  | 6.9 (-20.8 to 34.59) | 0.621 |
| PT | 1.09 (-0.377 to 5.95) | 0.659 |  | 2.4 (-3.08 to 7.89) | 0.386 |  | -10.77 (-36.17 to 14.62) | 0.401 |
| FO | 5.51 (0.51 to 10.51) | 0.031 |  | -1.31 (-6.62 to 3.99) | 0.623 |  | -32.66 (-58.7 to -6.62) | 0.015 |
| ***Prognostic indicators:*** |  |  |  |  |  |  |  |  |
| Age at baseline | 0.06 (-0.24 to 0.37) | 0.672 |  |  |  |  |  |  |
| Gender | -0.32 (-3.86 to 3.22) | 0.858 |  |  |  |  |  |  |
| Body Mass Index | 0.09 (-0.26 to 0.43) | 0.619 |  |  |  |  |  |  |
| Duration of knee pain | ***-0.08 (-0.15 to -0.01)*** | ***0.034*** |  | **-0.07 (-0.1 to -0.03)** | **0.000** |  | -0.11 (-0.19 to -0.02) | 0.013 |
| *Interaction FO+PT x Duration* | ***0.11 (0.01 to 0.2)*** | ***0.030*** |  |  |  |  | 0.11 (0 to 0.21) | 0.045 |
| *Interaction PT x Duration* | -0.04 (-0.13 to 0.05) | 0.417 |  |  |  |  | -0.05 (-0.15 to 0.05) | 0.368 |
| *Interaction FO x Duration* | 0.05 (-0.05 to 0.14) | 0.316 |  |  |  |  | 0.08 (-0.02 to 0.19) | 0.126 |
| VAS worst (baseline) | ***-0.11 (-0.22 to 0.01)*** | ***0.063*** |  |  |  |  |  |  |
| FIQ (baseline) | -0.09 (-1.9 to 1.73) | 0.925 |  |  |  |  | -2.02 (-3.99 to -0.04) | 0.046 |
| *Interaction F)+PT x FIQ* | -0.53 (-3.08 to 2.03) | 0.683 |  |  |  |  | -0.6 (-3.14 to 1.94) | 0.639 |
| *Interaction PT x FIQ* | ***2.45 (0.24 to 4.66)*** | ***0.030*** |  |  |  |  | 1.68 (-0.62 to 3.98) | 0.149 |
| *Interaction FO x FIQ* | ***2.05 (-0.4 to 4.51)*** | ***0.100*** |  |  |  |  | 2.75 (0.33 to 5.18) | 0.027 |
| KPS (baseline) | ***0.45 (0.27 to 0.63)*** | ***0.000*** |  | **0.33 (0.13 to 0.53)** | **0.002** |  | **0.4 (0.17 to 0.63)** | **0.001** |
| Arch height (study side) | -0.09 (-0.44 to 0.26) | 0.603 |  |  |  |  |  |  |
| Pain-free step downs (baseline) | 0.25 (-0.08 to 0.58) | 0.135 |  |  |  |  |  |  |
| **Adjusted R^2^** |  |  |  | **29.5%** |  |  | **49.7%** |  |
| **FUNCTIONAL INDEX QUESTIONNAIRE** | |  |  |  |  |  |  |  |
| Intercept |  |  |  | 6.76 (2.34 to 11.17) | 0.003 |  | 6.76 (2.34 to 11.17) | 0.003 |
| ***Treatment group (ref: Flat inserts):*** | |  |  |  |  |  |  |  |
| FO+PT | 0.05 (-1.14 to 1.23) | 0.939 |  | 0.29 (-1.07 to 1.66) | 0.672 |  | 0.29 (-1.07 to 1.66) | 0.672 |
| PT | 0.85 (-0.35 to 2.04) | 0.163 |  | 0.96 (-0.44 to 2.36) | 0.175 |  | 0.96 (-0.44 to 2.36) | 0.175 |
| FO | -0.51 (-1.68 to 0.66) | 0.389 |  | -0.12 (-1.48 to 1.24) | 0.865 |  | -0.12 (-1.48 to 1.24) | 0.865 |
| ***Prognostic indicators:*** |  |  |  |  |  |  |  |  |
| Age at baseline | ***0.19 (0.05 to 0.33)*** | ***0.009*** |  | 0.09 (0 to 0.17) | 0.051 |  | 0.09 (0 to 0.17) | 0.051 |
| *Interaction FO+PT x Age* | ***-0.24 (-0.45 to -0.04)*** | ***0.020*** |  |  |  |  |  |  |
| *Interaction PT x Age* | ***-0.25 (-0.45 to -0.05)*** | ***0.014*** |  |  |  |  |  |  |
| *Interaction FO x Age* | -0.08 (-0.29 to 0.13) | 0.453 |  |  |  |  |  |  |
| Gender | -0.5 (-1.34 to 0.34) | 0.240 |  |  |  |  |  |  |
| Body Mass Index | 0 (-0.08 to 0.09) | 0.953 |  |  |  |  |  |  |
| Duration of knee pain | ***-0.02 (-0.02 to -0.01)*** | ***0.000*** |  | **-0.02 (-0.03 to -0.01)** | **0.000** |  | **-0.02 (-0.03 to -0.01)** | **0.000** |
| VAS worst (baseline) | ***-0.04 (-0.06 to -0.01)*** | ***0.004*** |  |  |  |  |  |  |
| FIQ (baseline) | ***0.4 (0.21 to 0.59)*** | ***0.000*** |  | -0.07 (-0.37 to 0.23) | 0.656 |  | -0.07 (-0.37 to 0.23) | 0.656 |
| KPS (baseline) | ***0.1 (0.06 to 0.14)*** | ***0.000*** |  | 0.07 (0.01 to 0.14) | 0.024 |  | 0.07 (0.01 to 0.14) | 0.024 |
| Arch height (study side) | -0.03 (-0.11 to 0.06) | 0.552 |  |  |  |  |  |  |
| Pain-free step downs (baseline) | ***0.1 (0.02 to 0.18)*** | ***0.013*** |  | 0.09 (-0.01 to 0.18) | 0.073 |  | 0.09 (-0.01 to 0.18) | 0.073 |
| **Adjusted R^2^** |  |  |  | **26.6%** |  |  | **26.6%** |  |

^ n = 170 (worst pain VAS), n = 145 (AKP Scale), n = 171 (FIQ); n less than 179 due to missing data

FO+PT foot orthoses plus physiotherapy; PT physiotherapy; FO foot orthoses

***Included in multivariate analysis (significant at p = 0.1)***. **Significant at *p* = 0.01**
